# The Prevalence of Anti-Aquaporin 4 Antibody in Patients with Idiopathic Inflammatory Demyelinating Diseases Presented to a Tertiary Hospital in Malaysia: Presentation and Prognosis

**DOI:** 10.1155/2017/1359761

**Published:** 2017-01-19

**Authors:** S. Abdullah, W. F. Wong, C. T. Tan

**Affiliations:** ^1^Division of Neurology, Department of Medicine, University Malaya Medical Centre, Kuala Lumpur, Malaysia; ^2^Department of Medical Microbiology, University Malaya Medical Centre, Kuala Lumpur, Malaysia

## Abstract

*Background*. There have been inconsistent reports on the prevalence and pathogenicity of anti-Aquaporin 4 (AQP4) in patients presented with idiopathic inflammatory demyelinating diseases (IIDDs).* Objective*. To estimate the prevalence of anti-AQP4 antibody in patients with IIDDs presented to University Malaya Medical Centre in terms of patients' clinical and radiological presentations and prognoses.* Methods*. Retrospective data review of IIDDs patients presented from 2005 to 2015. Patients were classified into classical multiple sclerosis (CMS), opticospinal (OS) presentation, optic neuritis (ON), transverse myelitis (TM), brainstem syndrome (BS), and tumefactive MS. Anti-Aquaporin 4 antibody was tested using the Indirect Immunofluorescence Test (IIFT) cell-based assay. Statistical analysis was done using the SPSS version 20.* Results*. Anti-AQP4 antibody was detected in 53% of patients presented with IIDDs. CMS was more common in the seronegative group, 27/47 (57.45%; *p* < 0.001). Conversely, OS involvement was more common in the seropositive group, 26/53 (49.06%; *p* < 0.001). Longitudinally extensive spinal cord lesions (LESCLs) on MRI were also more common in the seropositive group, 29/40 (72.50%; *p* = 0.004). Only 2/40 (5.00%) had MRI evidence of patchy or multiple short-segment spinal cord lesions in the AQP4-positive group (*p* = 0.003). The relapse rate and Expanded Disability Status Scale (EDSS) were also higher in the seropositive group (5.43 versus 3.17, *p* = 0.005; 4.07 versus 2.51, *p* = 0.006, resp.). Typical clinical presentations that defined NMO were also seen in the seronegative patients, but in a lower frequency.* Conclusion*. Our cohort of patients had a higher prevalence of seropositivity of anti-AQP4 antibody as compared to those in Western countries. This was also associated with a more typical presentation of opticospinal involvement with LESCLs on MRI, a higher rate of relapse, and EDSS.

## 1. Introduction

Idiopathic inflammatory demyelinating diseases (IIDDs) of the central nervous system include a broad spectrum of neurological presentations. Although they can be differentiated based on the clinical, imaging, and laboratory findings, significant overlaps could lead to diagnostic uncertainty. The relapsing form of multiple sclerosis (MS) is considered the most common IIDD [1, 2]. However, in the last decade, the entity of neuromyelitis optica (NMO) has been subjected to multiple reviews that lead to the declaration of a new nomenclature known as neuromyelitis optica spectrum disorder (NMOSD) as a separate disease entity [3–5]. Previously limited to opticospinal involvement and seropositivity of anti-Aquaporin 4 (AQP4) or NMO IgG antibody, NMOSD also includes clinical syndromes and MRI findings related to the area postrema, other brainstem, diencephalic, or cerebral presentations in addition to seronegative opticospinal cases with optic chiasmal involvement and longitudinally extensive spinal cord lesions (LESCLs) [5]. The distinction between MS and NMOSD is crucial as it has a significant implication on the option for disease-modifying treatment [6].

MS is not common in Asia, with an estimated prevalence ranging between 1 and 2/100,000 in China to 7.7/100,000 in Japan [7]. Based on a 2008 review, the estimated prevalence in Malaysia was 2-3/100,000, which includes a mixed group of classical and opticospinal presentations with no known AQP4 status [8]. On the other hand, it is postulated that the relative frequency of NMOSD to that of MS is higher in Asia, ranging from 0.29 to 0.59 in Japan to 1.4 in Thailand as compared to 0.024 in Europe and 0.073 to 0.26 in Latin America [9]. However, there have been inconsistent reports on the prevalence of AQP4 seropositivity in the Korea and China studies [9, 10]. The aim of this study is to estimate the prevalence of anti-Aquaporin 4 antibody in patients with idiopathic inflammatory demyelinating diseases presented to University Malaya Medical Centre, Kuala Lumpur, Malaysia, in terms of their clinical and radiological presentations and prognoses.

## 2. Methodology

This was a retrospective study looking at patients presented to the neurology unit in the University Malaya Medical Centre, Kuala Lumpur, Malaysia, from 2005 till 2015, with IIDDs defined as the following:

1. Classical multiple sclerosis according to McDonald's 2005 and 2010 criteria
2. Opticospinal (OS) presentation
3. Monosymptomatic or recurrent optic neuritis
4. Monosymptomatic or relapsing transverse myelitis
5. Brainstem syndromes such as internuclear ophthalmoplegia and persistent hiccup/vomiting with corresponding MRI changes
6. Tumefactive MS

Data was obtained from medical records and the radiology department by a neurologist (SA). All patients were tested for connective tissue diseases, anti-phospholipid syndrome, thyroid antibody, B12 deficiency, and infective screening for retroviral disease, syphilis, and hepatitis B and C to rule out alternative diagnoses. Being a retrospective study, MRI at disease onset or the earliest imaging available was used for analysis. Longitudinally extensive spinal cord lesions (LESCLs) were defined as continuous lesions extending more than three vertebral segments, while lesions of fewer than three vertebral segments were defined as short or patchy spinal cord lesions. Relapse rates (RRs) were calculated based on the total number of relapses over the course of the disease, and the disability status was scored at the last follow-up in a stable disease phase using the Expanded Disability Status Scale (EDSS) of Kurtzke. One hundred patients were tested for anti-Aquaporin 4 (AQP4) antibody during the clinic follow-up, regardless of their relapse status, using the Indirect Immunofluorescence Test (IIFT) cell-based assay (EUROIMMUN IIFT, Germany). Two patients were excluded from the analysis due to the unavailability of AQP4 status.

Statistical analysis was done using the SPSS version 20. Descriptive data was expressed as total numbers and percentages; elsewhere, parametric data was analyzed using Student's *t*-test and nonparametric data using the Chi-square test. Values of *p* < 0.05 were considered statistically significant.

## 3. Results

### 3.1. Demographic Features

**Figure 1:**
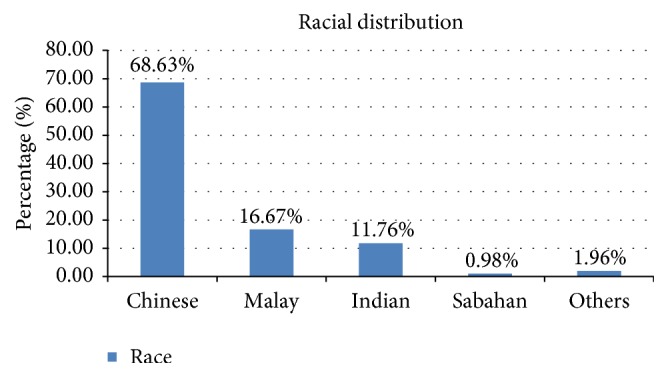


Patients' demographics were shown in Tables 1 and 2. In total, there were 102 patients presented to our centre with the diagnosis of IIDD from 2005 to 2015. The median follow-up was 11.4 years, ranging from 1 to 28 years. The Chinese were the predominant ethnic group affected, 70/102 (68.63%), followed by the Malay, 17/102 (16.67%), Indians, 12/102 (11.76%), the Sabahan, 1/102 (0.98%), and the Myanmarese, 2/102 (1.96%) (Figure 1). Regardless of the AQP4 status, both groups showed a higher female-to-male ratio of 12 : 1 in the seropositive cohort and about 4 : 1 in the seronegative group. There was a statistically significant difference in the age of onset, where the seropositive group demonstrated an older age of onset of 37.79 years as compared to 31.74 years in the seronegative group. The oldest patient in our cohort was 76 years old who presented with seropositive recurrent transverse myelitis with LESCLs on MRI. All the screening for alternative causes—in particular, underlying malignancy in view of her advanced age—was negative. Unfortunately, she had a poor response to high-dose methylprednisolone, plasma exchange, and immunosuppressive therapy. Nevertheless, late-onset NMOSD has been reported involving patients aged above 75 years, the oldest being 90 years of age, hence emphasizing the need to consider the diagnosis of NMOSD in elderly patients with classical long extensive transverse myelitis [11, 12].

### 3.2. Clinical Presentation (Figure 2)

Relapsing remitting disease was the most common disease course (77/102, 75.49%), with 25/102 (24.51%) presented as the first neurological deficit. There was a statistically significant difference in the clinical presentation between the AQP4 groups in which CMS was more common in the seronegative group, 27/47 (57.45%) as compared to 1/53 (1.89%) in the seropositive counterpart (*p* < 0.001). One seropositive patient presented with cortical symptoms of hemiparesis associated with diplopia, with corresponding T2 hyperintensity in the periventricular parietal and frontal white matter. On the other hand, OS involvement was more common in the seropositive group as compared to the seronegative group, 26/53 (49.06%) versus 5/47 (10.64%; *p* < 0.001). Isolated ON was almost equally distributed in both groups, 4/53 (7.55%) in the seropositive group versus 4/47 (8.51%) in the seronegative group. TM and BS involvement were more frequent in the seropositive groups, TM 16/53 (30.19%) versus 9/47 (19.15%), brainstem syndrome and opticospinal (BSOS), 3/53 (5.66%) versus 1/47 (2.13%), and brainstem syndrome and transverse myelitis (BSTM), 2/53 (3.77%) versus 0/47. However, they did not reach statistical significance (Table 2).

### 3.3. AQP4 and Cerebral Spinal Fluid (CSF) Oligoclonal Band (OCB) Status

Data on CSF OCB was only available in 12/53 patients in the AQP4-positive group and 30/47 patients in the seronegative group. CSF OCB was detected in 7/12 (58.33%) patients in the seropositive group and 17/30 (56.67%) patients in the seronegative group (Table 2). Nevertheless, 13 patients were negative for both AQP4 and CSF OCB.

### 3.4. Spinal Cord Lesion on the MRI

The MRI of the spinal cord was available in 40/53 AQP4-positive patients and in 30/43 AQP4-negative patients. In total, 29/40 (72.50%) in the seropositive group had longitudinally extensive spinal cord lesions (LESCLs) on MRI as compared to 7/37 (18.92%) in the seronegative group, with a *p* value of 0.004. In contrast, only 2/40 (5.00%) had MRI evidence of patchy or multiple short-segment spinal cord lesions in the AQP4-positive group as compared to 19/37 (51.35%) in the AQP4-negative group. This difference in spinal cord involvement also reached a statistical significance with a *p* value of 0.003. Only 3/29 patients with LESCLs in the seropositive group had involvement of the cervicomedullary junction (CMJ). However, there was no significant difference in terms of CMJ involvement between the two groups (Table 2). Two seropositive patients had LESCLs involving the conus medullaris. This was not observed in the seronegative group.

### 3.5. Relapse Rates and EDSS

Patients in the seropositive group demonstrated a significantly higher rate of relapse (mean RR 5.43 versus 3.17; *p* = 0.005) and EDSS (median 3.5 [range 0.0–9.5] versus 1.0 [range 0.0–9.5]; mean 4.07 versus 2.51; *p* = 0.006) as compared to the seronegative group (Table 3).

## 4. Discussion

Serum anti-AQP4 antibody was detected in 53% of patients in this study. This was higher than the previously reported frequency in the Thai, Japanese, and Korean cohorts that ranges from 33% to 39% [7, 13, 14]. Nevertheless, this supports the proposal that NMOSD is more common in the Asian region, emphasizing the role of differences in genetic make-up. The most common presentation in this seropositive group was opticospinal involvement (49.06%; *p* < 0.004) followed by transverse myelitis (30.19%) and optic neuritis (7.55%). In total, this made up 86.80% of all the presentation perceived to be characteristic of NMOSD [3–5]. Brainstem presentation, with or without optic nerve and/or spinal cord involvement, was seen in 5/53 (9.43%) patients in the seropositive group as compared to 1/47 (2.13%) in the seronegative group. The clinical presentation of brainstem involvement was different between the two groups, where the AQP4-positive patients presented with persistent vomiting or hiccups as opposed to internuclear ophthalmoplegia in the seronegative group. This was consistent with the clinical spectrum of NMOSD [5, 15]. However, the MRI findings were not restricted to the area postrema, but a more extensive involvement of the brainstem (pontine-medullary [2/5] or cervicomedullary [3/5]).

**Figure 3:**
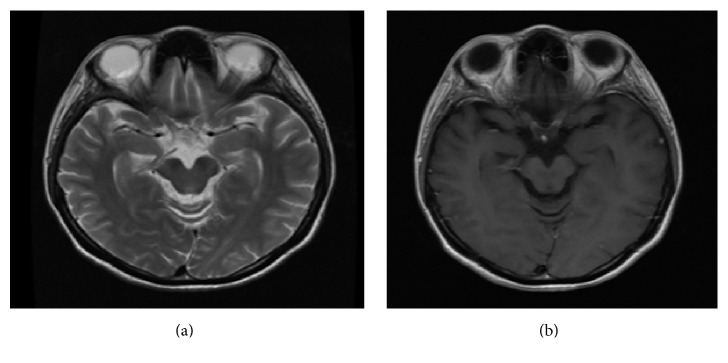
(a) T2 weighted image showing bilaterally oedematous optic nerves and optic chiasma with (b) patchy enhancement of the optic chiasma on T1 weighted image. This patient was negative for both anti-AQP4 antibody and CSF OCB.

In the seronegative group, the most common presentation was CMS (57.45%; *p* < 0.001) followed by transverse myelitis (19.14%), opticospinal involvement (10.64%), and optic neuritis (8.51%). Even though the frequency of optic neuritis was marginally higher as compared to the seropositive group, it was not statistically significant; hence, it was not a defining clinical presentation that differentiates between MS and NMOSD. In total, nine patients presented with bilateral optic neuritis, four seronegative and five seropositive. However, chiasmal involvement was seen in only one patient who was negative for both anti-AQP4 antibody and CSF OCB (Figure 3). Chiasmal ON is a rare condition that is mostly associated with inflammatory demyelinating disorders, such as NMOSD and to a lesser extent MS, once a suprasellar compressive lesion has been ruled out. Even though favorable prognoses in terms of recovery of visual acuity are reported in cases of idiopathic chiasmal optic neuritis, those with NMOSD usually end up with severe and irreversible visual impairment [16].

The reported rate of OCB detection in the seropositive group ranged from 16.4% to 35%. When present, it was transient and associated with acute relapses [17, 18]. One of the interesting findings in our study was the higher rate of CSF OCB detection in the AQP4-positive group of up to 58.33%. However, this outcome might be a reflection of a small sample size (7/12) rather than a true indication of an increased intrathecal IgG synthesis.

**Figure 4:**
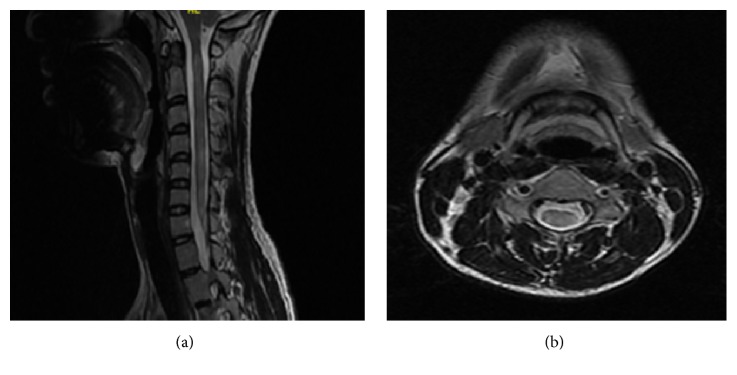
Sagittal (a) and axial (b) T2 weighted images of LESCLs with cord oedema in a patient with negative anti-AQP4 antibody.

In the subgroup of patients presented with acute transverse myelitis with or without optic neuritis, a similar trend of previously described LESCLs was observed in the seropositive group. Nevertheless, up to 18.92% of seronegative patients also presented with LESCLs associated with cord oedema (Figure 4). This was also observed in the Korean, Thai, and Japanese studies, suggesting heterogeneity of AQP4 autoimmunity [9, 13, 19]. It was postulated that AQP4-negative patients with clinical characteristics of NMO might be positive for anti-myelin oligodendrocyte glycoprotein (MOG) antibody. Of interest, there are fundamental differences in the pathogenesis and clinical features of AQP4-positive NMOSD and MOG-Ig-positive AQP4 seronegative phenotype. Seropositive NMOSD is an autoimmune astrocytopathy with AQP4 identified as the target autoantigen. On the other hand, MOG is produced by the oligodendrocytes and is recognized as the target autoantigen candidate in acute disseminated encephalomyelitis (ADEM) and a selective subgroup of adult type II MS (antibody-mediated demyelination) [20]. MOG-IgG positivity is also known to be serologically unstable and can be undetectable in more than 50% of previously seropositive patients; hence, it has a possible role as a marker of myelin injury in the acute phase rather than as a distinct autoantigen target [21]. In terms of clinical presentation, anti-MOG antibody-positive patients were noted to have a younger age of onset, more restricted phenotype with preference for optic nerve rather than spinal cord, and higher male-to-female ratio and were less likely to relapse [22–25]. This antibody was not tested in our subgroup of patients who were negative for both AQP4 antibody and CSF OCB. Nevertheless, besides the younger age of onset (mean 26.71 years, range 6 to 35), female predominance and recurrent events were noted in 5/7 (71.43%) of our study cohort with a mixture of CMS (5/13), ON (1/13), TM (5/13), and OS (2/13) presentations. Hence, the role of anti-MOG antibody in the pathogenesis of seronegative NMOSD remained to be explored [26].

There was a similar trend of higher relapse rates and EDSS in seropositive patients. This was likely due to a more extensive spinal cord and optic nerve involvement in accordance with the great abundance of AQP4 receptors in the optic nerve and spinal cord [27, 28]. Nevertheless, the patient with the highest EDSS score of 9.5 had a total of seven relapses with recurrent optic neuritis and long-segment transverse myelitis, a poor response to both high-dose corticosteroid and plasma exchange, and was negative for both the anti-AQP4 antibody and CSF OCB.

In conclusion, our cohort of patients has a higher prevalence of seropositivity of anti-AQP4 antibody as compared to the Western countries. This was also associated with a more typical presentation of opticospinal involvement with LESCLs on MRI. A similar pattern was also observed in Japan, Thailand, and Korea. AQP4 seropositivity was also associated with a higher rate of relapse and EDSS. Although the clinical and imaging presentation of AQP4-positive NMOSD patients appeared to be more well defined, there were still overlaps in terms of clinical presentation and imaging findings in the AQP4-negative patients. Hence, the entity of seronegative NMOSD and/or the role of anti-MOG antibody need to be further explored. We acknowledge the limitations of this study, which is retrospective and descriptive in nature. However, this is the first study done in Malaysia comparing the NMOSD and MS groups in terms of their presentations and prognoses.

## Figures and Tables

**Figure 2:**
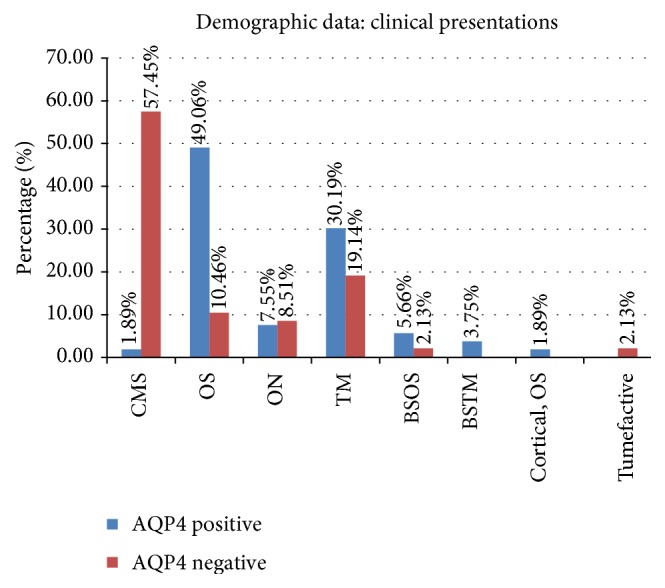


**Table 1:** 

Race	Number (*n*)	Percentage (%)
Chinese	70	68.63%
Malay	17	16.67%
Indian	12	11.76%
Sabahan	1	0.98%
Others	2	1.96%

Total	102	100%

**Table 2:** 

		AQP4 positive (*n* = 53)	AQP4 negative (*n* = 47)	*p* value
Number of M/F		4/49 (1 : 12.25)	10/37 (1 : 3.70)	
Age of onset (years)		37.79 (±SD 13.064)	31.74 (±SD 12.669)	0.021^*∗*^
Frequency of symptoms				
CMS		1/53 (1.89%)	27/47 (57.45%)	<0.001^*∗*^
Tumefactive		0/53 (0.00%)	1/47 (2.13%)	0.323
ON		4/53 (7.55%)	4/47 (8.51%)	0.861
TM		16/53 (30.19%)	9/47 (19.15%)	0.140
OS (ONTM)		26/53 (49.06%)	5/47 (10.64%)	<0.001^*∗*^
BSOS		3/53 (5.66%)	1/47 (2.13%)	0.373
Cortical, OS		1/53 (1.89%)	0/47 (0.00%)	0.349
BSTM		2/53 (3.77%)	0/47 (0.00%)	0.159
CSF OCB				
Positive		7/12 (58.33%)	17/30 (56.67%)	
Negative		5/12(41.67%)	13/30 (43.33%)	
NA		41/53 (77.36%)	17/47 (36.17%)	
LESCLs (>3VB)		26/40 (65.00%)	6/37 (16.22%)	0.025^*∗*^
CMJ + LESCLs		3/40 (7.50%)	1/37 (2.70%)	0.696
Total LESCLs		29/40 (72.50%)	7/37 (18.92%)	0.004^*∗*^
CMJ		1/40 (2.50%)	1/37 (2.70%)	0.704
Patchy, multiple short segments		2/40 (5.00%)	19/37 (51.35%)	0.003^*∗*^
Normal MRI spine		4/40 (10.00%)	10/37 (27.03%)	0.466
MRI spine NA		10/53 (18.87%)	13/47 (27.66%)	

M = male, F = female, AQP4 = Aquaporin 4, SD = standard deviation, CMS = classical multiple sclerosis, ON = optic neuritis, TM = transverse myelitis, ONTM = optic neuritis and transverse myelitis, OS = opticospinal, BSOS = brainstem and opticospinal, BSTM = brainstem and transverse myelitis, CSF OCB = Cerebral Spinal Fluid Oligoclonal Band, NA = not available, LESCLs = longitudinally extensive spinal cord lesions, VB = vertebral body, and CMJ = cervicomedullary junction. ^*∗*^Statistically significant with a *p* value < 0.05.

**Table 3:** 

	AQP4 positive	AQP4 negative	*p* value
Relapse			
Mean	5.43 (±SD 4.357)	3.17 (±SD 3.384)	0.005^*∗*^
EDSS			
Mean	4.07 (±SD 2.867)	2.51 (±SD 2.675)	0.006^*∗*^
Median	3.5 (range 0.0 to 9.5)	1.0 (range 0.0 to 9.5)	

AQP4 = Aquaporin 4, EDSS = Expanded Disability Status Scale, and SD = standard deviation. ^*∗*^*p* value < 0.05, hence, statistically significant.
